# Using PhotoVoice to understand mindfulness in health care practitioners

**DOI:** 10.4102/hsag.v27i0.1942

**Published:** 2022-09-28

**Authors:** Iram Osman, Veena Singaram

**Affiliations:** 1 Department of Clinical Medicine, Faculty of Health Sciences, University of KwaZulu-Natal, Durban, South Africa

**Keywords:** COVID-19, health professionals, mindfulness, PhotoVoice, self-care

## Abstract

**Background:**

The disruptions of the coronavirus disease 2019 (COVID-19) pandemic have placed added stress on health care practitioners’ (HCPs) mental health. Mindfulness-based interventions (MBIs) have been reported to increase the awareness of burnout and promote self-care practices that enhance mental well-being.

**Aim:**

To gain insight into the use of mindfulness through the lens of PhotoVoice on how HCPs reflected on their stressors and sense of self whilst working as frontline workers during the COVID-19 pandemic.

**Setting:**

This study was conducted online with HCPs working in South Africa during the first wave of COVID-19.

**Method:**

A four-week MBI intervention was implemented using Zoom. An exploratory qualitative analysis was conducted using a PhotoVoice methodology. Interpretative phenomenological analysis was used to generate themes. Fifty-five HCPs consented to participate in this study.

**Results:**

The major themes identified were operating on autopilot, feeling a sense of overwhelm because of COVID-19, using faith to cope and being able to attain a sense of self-compassion by the end of the intervention.

**Conclusions:**

Using visual representations, HCPs were able to share the development of their reperceived lived experiences of increased self-compassion as they navigated the dilemmas and disruptions of the pandemic.

**Contribution:**

A brief online MBI was impactful enough to show a reappraisal of the stressors of COVID-19, such that HCPs felt calmer, more competent and more compassionate. PhotoVoice methodology is recommended for future studies and mindfulness courses. It facilitates a deeper understanding of the practice of imbuing mindfulness and its impact on stressors and the self.

## Introduction

A health care practitioner’s (HCP) duties are usually demanding; however, the current coronavirus disease 2019 (COVID-19) pandemic has made the work overwhelming (Rees et al. 2018; Shaukat, Ali & Razzak 2020). Health care practitioners face many challenges during the COVID-19 pandemic, leading to feelings of powerlessness and burnout (Osman, Hamid & Singaram 2021). Most frontline HCPs in this study were within the moderate perceived stress levels and experienced emotional exhaustion (a subscale of burnout) (Osman et al. 2021). The elevated stress levels were related to the lack of resources, uncertainty around the condition, fear of contagion, having to lend emotional support to dying patients, higher death rates and feeling unsupported by employers (Fernandez et al. 2020; Osman et al. 2021). These factors were intensified by the lack of access to healthy coping mechanisms like visiting the gym, prayer meetings and social support. These restrictions were because of the national lockdowns to prevent the spread of the disease (Osman et al. 2021).

The first COVID-19 case in South Africa was identified on 05 March 2020. Ten days later, a state of disaster was declared in the country. Lockdown level 3 was implemented at the time of the study. The first wave of infections of COVID-19 lasted from week 24 (mid-June 2020) to week 34 (last week of August 2020) (Giandhari et al. 2020), during which infection and mortality rates of HCPs were high (Lambert et al. 2020), putting HCPs at a higher risk for burnout.

Burnout is a syndrome consisting of emotional exhaustion, depersonalisation and reduced personal accomplishment resulting from chronic workplace stress in professionals exposed to high-pressure situations (Chirico, Nucera & Magnavita 2021). COVID-19 exacerbated symptoms of burnout across the world because of the unprecedented demands on HCPs, often beyond their capacities (Leo et al. 2021). Current research on burnout amongst HCPs during COVID-19 (Callus et al. 2020; Søvold et al. 2021; Sultana et al. 2020) highlighted the need to adopt evidence-based approaches to encourage self-care practices to promote mental well-being and reduce stress levels in HCPs. One of the interventions Sultana et al. (2020) recommended was mindfulness-based interventions (MBIs). Mindfulness is a Buddhist concept that has been secularised and is taught globally as a form of mental training. Mindfulness develops a sense of awareness and observation of thoughts and emotions. It enhances the modulation of one’s behaviour to rise above self-focused needs and narratives to increase prosocial behaviour (DeMauro et al. 2019; Vago & Silbersweig 2012).

The theoretical framework for this paper was positive reappraisal that was linked to both phenomenology and mindfulness. Positive reappraisal is a critical part of the stress response, especially when coping with adversity. According to Folkman and Moskowitz (2000), it is not the situation that triggers the stress response but the way the stressor is perceived and one’s competence appraised to handle it. There is a primary appraisal of the situation, and if a stimulus is seen as harmful, the stress response is activated, and one experiences distress (Folkman & Moskowitz 2000). However, in a secondary reappraisal, the individual’s coping mechanism is assessed. If the person feels competent enough to handle it, the stress response and distress experienced reduce (Garland, Gaylord & Park 2009). Hence, positive reappraisal works in two ways: a reinterpretation (seeing things differently) and distancing (seeing things more objectively) (Torre & Lieberman 2018). Mindfulness involves the process of decentring, which involves both distancing and subsequent reinterpretation, leading to a shift in awareness that enables alternate perceptions of life events. This study focuses on enhancing positive reappraisal by increasing awareness and a sense of objectivity by using pictures to make thoughts and emotions overt.

DeMauro et al. (2019), in an interdisciplinary review of mindfulness research in caring professions, found that mindfulness enhanced professional practice through mechanisms of therapeutic presence and self-care. It was, however, recommended to explore the processes and outcomes of how mindfulness works inter- and intrapersonally. Mahalingam and Rabelo (2019) reported similar findings on the benefits of MBIs in terms of compassion, mental well-being and emotional regulation, leading to both personal and social transformations. However, they recommended more phenomenological methods such as PhotoVoice to understand how mindfulness was a process and influenced participants’ self-perceptions.

There is a need for research on the perspectives of frontline workers during the pandemic and to give voice to their everyday challenges (Sackett & Jenkins 2015). PhotoVoice is a visual arts–based methodology (Wang & Burris 1994) where participants are encouraged to capture a point in time through a photograph. It is known to be a research tool that empowers participants by allowing them the opportunity to express issues that are salient to them (Mahalingam & Rabelo 2019). PhotoVoice is a user-friendly method of data collection gaining popularity, as it can tap into emotions and perceptions the way text cannot (Liebenberg et al. 2018).

Very few studies have used phenomenological approaches to study the effectiveness of mindfulness (Mahalingam & Rabelo 2019). There is also limited PhotoVoice exploration in MBI studies (Ciolan & Manasia 2017), especially during a health care crisis like COVID-19. This study aims to gain insight into the use of mindfulness through the lens of PhotoVoice on how HCPs reflected on their stressors and sense of self whilst working during the COVID-19 pandemic.

## Methods

### Study design

A qualitative approach was adopted for this study. Data were collected using PhotoVoice. Interpretative phenomenological analysis (IPA) was used to analyse the data. The use of PhotoVoice within phenomenological inquiry provided direction and purpose regarding the experience of reflection and positive reappraisal initiated by the MBI. Interpretative phenomenological analysis was chosen because of the depth of analysis that involved the double hermeneutic of participants endeavouring to make sense of their experiences and the researcher seeking to actively understand how they did so (Smith & Osborn 2008). Interpretative phenomenological analysis and mindfulness focus on the operation of the mind through first-person perception by closely observing one’s subjective and sensory experiences.

PhotoVoice is a research method in which participants capture their lived experiences in pictures, which researchers then use as data (Langley-Brady 2019). PhotoVoice can capture participants’ perspectives through the combined use of imagery and narrative, which fosters authentic discussions that stimulate change (Ciolan & Manasia 2017). The framework followed by this study is the one initially proposed by Wang and Burris (1994), as outlined in the following steps (Ciolan & Manasia 2017):

1. Select the research problem that requires exploration: how did the weekly mindfulness exercises and training influence the HCPs’ sense of self during the COVID-19 pandemic?
2. Recruit participants: invites were shared to HCPs via social media platforms.
3. PhotoVoice group meetings: because of lockdown restrictions, this was replaced by creating a group discussion using mobile phones via WhatsApp messenger, an American freeware programme for instant messaging and voice-over-IP service.
4. Collect data: images and explanations were shared during the four-week MBI via WhatsApp.
5. Analyse data using IPA.

### Mindfulness intervention

#### Setting

The study was conducted with HCPs working in the public health care system in South Africa during the first wave of the COVID-19 pandemic. Almost half of the South African population resides in rural areas with limited facilities and a considerable treatment gap of only 12% of attending HCPs (Besada et al. 2020). Eighty-three per cent of the entire South African population relies on the crumbling public health care system, which is underfunded and poorly managed (Ngobeni, Breitenbach & Aye 2020). The delivery of health services had challenges across the provinces relating to budget constraints, poor maintenance of existing health care facilities, the unavailability of specialised staff and a high level of medicolegal claims resulting in decreased resources for health care access (Ngobeni et al. 2020).

#### Study population and sampling strategy

Purposive snowball sampling was used (Naderifar, Goli & Ghaljaei 2017). The information and link to register for the study were posted on interns’, medical registrars’ and HCPs’ social media platforms such as WhatsApp groups and Facebook pages. Registered HCPs were encouraged to share the link with colleagues. The study population was HCPs over the age of 18 working in South Africa during COVID-19. Consent forms and registration details were provided to interested participants via SurveyMonkey (http://www.surveymonkey.com). Sixty-five HCPs showed interest in participating, but only 55 met all criteria and attended all four sessions. The inclusion criteria included being over 18 years old, registered as an HCP in South Africa and currently working in South Africa during COVID-19. Health care practitioners working outside of SA were excluded from the study. The sample consisted of 23 medical doctors, 12 psychologists, eight physiotherapists, seven occupational therapists, two radiographers, one podiatrist, one chiropractor and one dentist. The age of participants ranged from 23 to 63 years, and the participants were located across four provinces of South Africa, namely KwaZulu-Natal, Gauteng, Limpopo and the Western Cape.

### Data collection

A shortened version of a standard 8-week mindfulness-based stress reduction programme (Watkin n.d.) was adopted and conducted online over four weeks during the first wave of COVID-19 in South Africa. The intervention consisted of weekly one hour group sessions using the online platform Zoom, conducted by two qualified mindfulness teachers and facilitated by the researchers. Each session consisted of a teaching section and experiential exercises to teach participants how to understand and apply mindfulness in their lives, as outlined in Table 1.

**TABLE 1:** Summary of 4-week mindfulness-based interventions.

Week	Mindfulness-based interventions
**Week 1**	3-min breathing space Raisin exercise Body scan Input on mindlessness and mindfulness
**Week 2**	3-min breathing space Yoga Input on breath and body awareness Walking meditation
**Week 3**	3-min breathing space Awareness of thoughts Input on stress, acceptance, allowing and letting be 3-min breathing space and action
**Week 4**	3-min breathing space Body scan Input on empathy and self-care Lovingkindness meditation

*Source:* Watkin, M., n.d., *Mindfulness-based cognitive therapy 4*, unpublished manual

**FIGURE 1:**
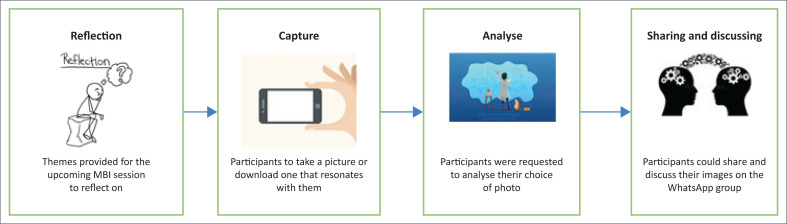
A schematic representation of data collection using PhotoVoice.

Figure 1 provides a schematic representation to explain the Photovoice process. Participants were requested to send a picture every week – either taken by themselves using a camera or cell phone or found through a web search – illustrating their experience (14 July to 05 August 2020). The instruction was: ‘Please provide a picture that does not have identifiable features to describe your experiences when thinking of this week’s mindfulness practice’. This reason for specifically asking for no identifiable features was to ensure the confidentiality and anonymity of participants when reporting and disseminating research findings. Participants were also requested to analyse their choice of photo in each case. If desired, the participants could share their pictures on a WhatsApp group chat. This was encouraged to facilitate group discussion on WhatsApp. Although focus groups are the most popular method to collect data, alternative data collection methods can also be used (Pietkiewicz & Smith 2014). In this study, WhatsApp messages were used, which was convenient for the participants and ensured their safety during the COVID-19 pandemic.

### Data analysis and interpretation

The researchers interpreted the participants’ images by using IPA. The pictures and comments were pasted in separate Word documents for each week. Next, their experiences and explanations from the WhatsApp group chat were read and reread for in-depth understanding and then grouped into themes. Another way of aiding understanding was looking at each individual and noticing how this individual’s pictures and narrative developed over time. Both authors followed this process until a consensus was reached for verified themes. Bracketing of the researchers’ perceptions was achieved by confirming interpretations with the individual participants via WhatsApp. The overall themes and results were also shared with the whole group using a PowerPoint presentation. This helped to further verify the interpretation of the data. This was done at the one-month check-in after the intervention was completed (09 September 2020).

### Trustworthiness

There are some concerns regarding trustworthiness when using the PhotoVoice methodology. However, IPA is a dual interpretation process where the researcher tries to understand the participant who is also trying to understand themselves (Pietkiewicz & Smith 2014). To enhance this study’s rigour, direct quotations were used, and both authors looked through the data objectively and checked interpretation with the participants to validate the thematic analysis of the data presented. As both authors were aware of how their worldviews could influence the interpretation, each interpretation selected for this study was verified individually with the participants via WhatsApp. The themes were also verified with the whole group via Zoom. Furthermore, prior research and the theoretical framework of reappraisal guided the final analysis.

### Ethical considerations

Ethical approval was obtained from the University of KwaZulu-Natal Humanities and Social Science Research Ethics Committee (reference number: HSSREC/00000848/2019) and the KwaZulu-Natal Department of Health. Informed consent was obtained from participants prior to the study.

## Results

The findings were directly based on the HCPs’ experiences as reported by the participants in their own words and photographs. This ensured the credibility and dependability of the study. As mindfulness increased, the HCPs changed how they viewed themselves and subsequently related to their stressors. Initially, HCPs described themselves as being on autopilot to compensate against the feelings of overwhelm. Finding faith helped them to cope, leading finally to self-compassion.

### Autopilot

**FIGURE 2:**
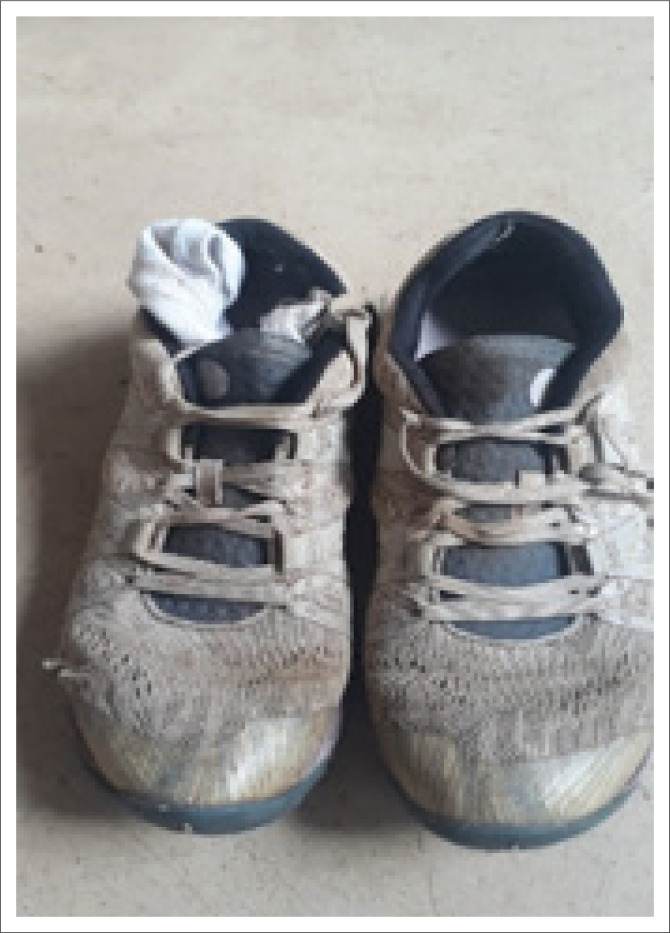
The participant photographed her worn-out pair of running shoes.

In the first week of the intervention, most participants sent pictures that alluded to feeling out of their bodies and not being present in the moment. The theme of autopilot entailed feeling rushed and inanimate, like a machine doing what is required without emotions. Pictures of remote controls, televisions, robots, factory lines, clocks and shoes were provided. Some participants perceived this as a coping mechanism to switch off and survive this time. The concept of just putting one foot in front of the other to push through was seen in Figure 2 where an experienced specialist family physician described a picture of worn-out running shoes in the following way: ‘It helped me realise that my autopilot is to walk, and in walking so much, I destroyed my shoes!’

**FIGURE 3:**
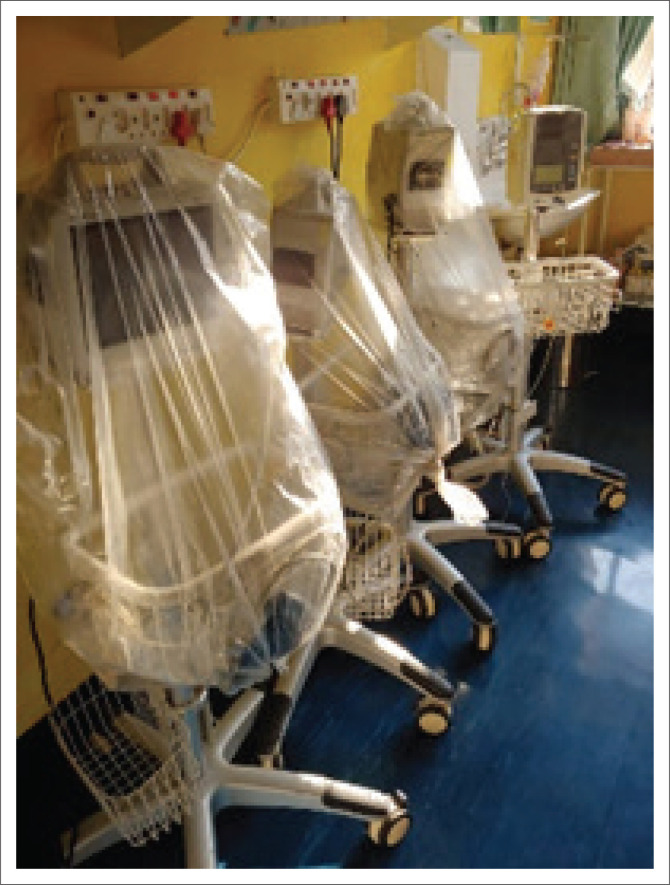
The participant photographed the electroencephalogram and vital signs monitors at the hospital where she works.

A young intern medical doctor working in a government facility described autopilot as (Figure 3):

‘So these are machines, the ones that we use to measure a patient’s vital signs. For me, it represents that robot and the ‘autopilot’ mode that I get into when I am at work and the monotony that we get into.’

From the state of autopilot, where the participants described machining through tasks whilst suppressing their emotions, the next step of the MBI was to create awareness of what participants were feeling, which is described in the next theme as overwhelm.

### Overwhelm

**FIGURE 4:**
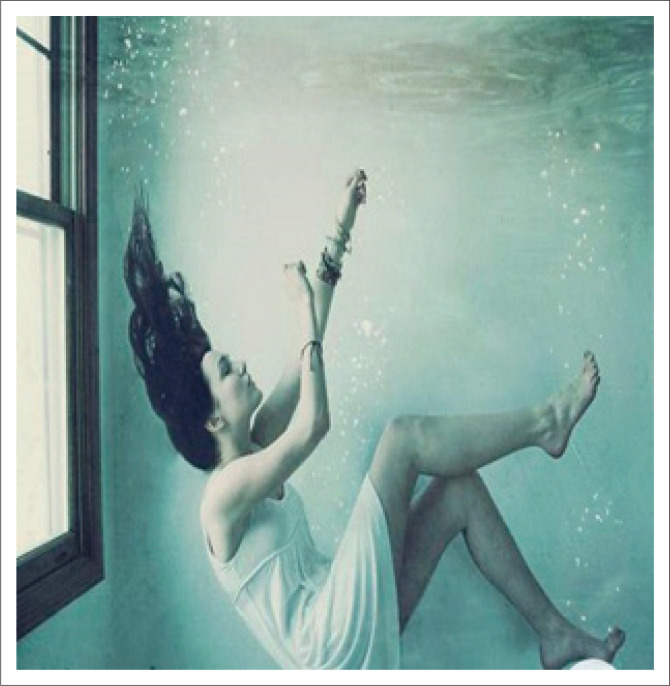
The participant downloaded a woman submerged in water.

In the second week, the pictures appeared to tell a story of a hectic period that elicited feelings of overwhelm. Participants reported feeling that they still had to maintain appearances of control despite feeling emotionally exhausted. The pictures sent under this theme were caricatures taken from the Internet. This inclination for parody illustrates how time constraints in which HCPs were trying to survive and keep up a brave front entailed so much effort that showing up with a genuine picture may have been too time-consuming for them. These high-stress levels were further observed in pictures of drowning and train wrecks. One such image is seen in Figure 4. A female intern medical doctor explained this picture, stating how it felt working in a government hospital during the COVID-19 pandemic:

‘I’m sure there are stresses other than COVID-19, but everything seems to have been drowned out by the panic around the pandemic …. Guilt about death and bereavement, becoming numb. Fear that I will contract COVID again (had it a couple weeks back and was very sick).’

Most HCPs felt overwhelmed because of being in the position to care for the health of others, especially when patients’ families could not be with them. This added more responsibilities to the HCPs without their support structures and coping mechanisms. Health care practitioners’ felt the need to keep up appearances that all was under control when, in fact, they felt desperate and helpless. An occupational therapist described this act as: ‘[*feels*] like I’m caught in a whirlwind whilst still trying to act like I have everything under control and continuing with work and life like normal’.

This comment highlighted how out of control everything felt at that time and the fear of letting go of this semblance of control. However, the next theme of the MBI was about focusing on faith and how it led to feelings of empowerment and making peace with the new normal.

### Faith

**FIGURE 5:**
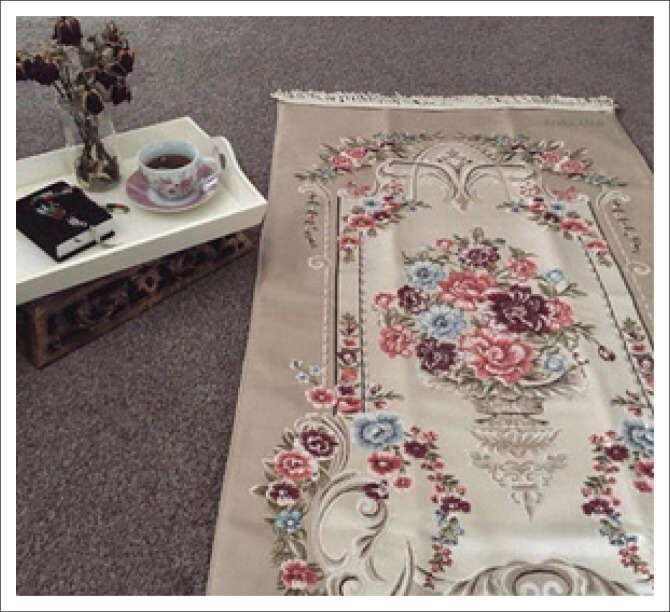
Participant photographed her Muslim prayer mat.

As the sessions progressed, a subtle change in participants’ statements emerged, with more enunciation of moments of calm and feelings of choice instead of helplessness. Faith was seen as a form of acceptance and surrender to a higher supernatural power in a challenging situation. This observation can be made as photos of prayer mats, Buddha and other prayer symbols were sent in by the participants (Figure 5). A Muslim participant, who works as a physiotherapist, explained:

‘Nothing brings more contentment and peace to my mind than turning to a higher power. In that moment, I’m relieved from all worldly stress; it brings mindfulness …. You become more aware of what you’ve been gifted and blessed with by the Creator, and all worldly troubles seem resolvable because I become more aware that life itself is a gift, and my blessings are countless.’

The concept of ‘letting be’ was important in this theme of faith and participants accepting their limitations and being grateful for their blessings during this time. There appeared to be a sense of relief, making way for HCPs to consider their own needs and engage in some self-care, which is discussed in the next theme of self-compassion.

### Self-compassion

**FIGURE 6:**
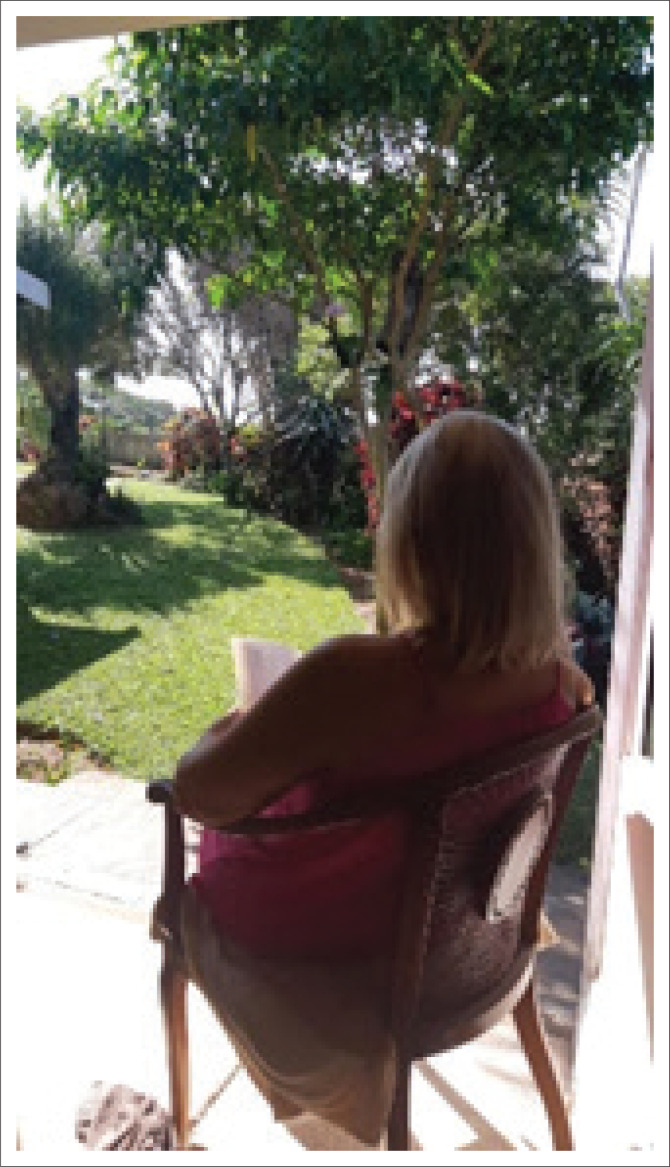
Participant’s photograph of sitting on a chair facing the garden and reading.

This theme focused on changing the relationship to self by making participants aware of how they treated themselves. The HCPs’ attitudes towards themselves changed by being more compassionate towards their own needs, translating into simple acts of joy. A trauma counsellor, who initially sent in a picture of a lady holding her head in her hands to express how she was ‘overwhelmed by anxiety’, had a changed mindset in the fourth week of practising mindfulness. As illustrated in Figure 6, she is sitting with her head held upright, reading a book and describing her current state as:

‘I am feeling much better. I can sit still in the sun and do nothing. I can read a novel. I walk slowly. I watch TV with my husband. I don’t beat myself up …. Your course has helped me realise I am loved. I am loving. I belong wherever I choose to belong. I can now thank my anxiety, as it is only trying to protect me. I thank it and I know I can control my anxiety. I don’t want fear to rule my life. I don’t want to rush from one place to another. I want to sit and breathe and feel the earth under my feet. I want to be heard.’

**FIGURE 7:**
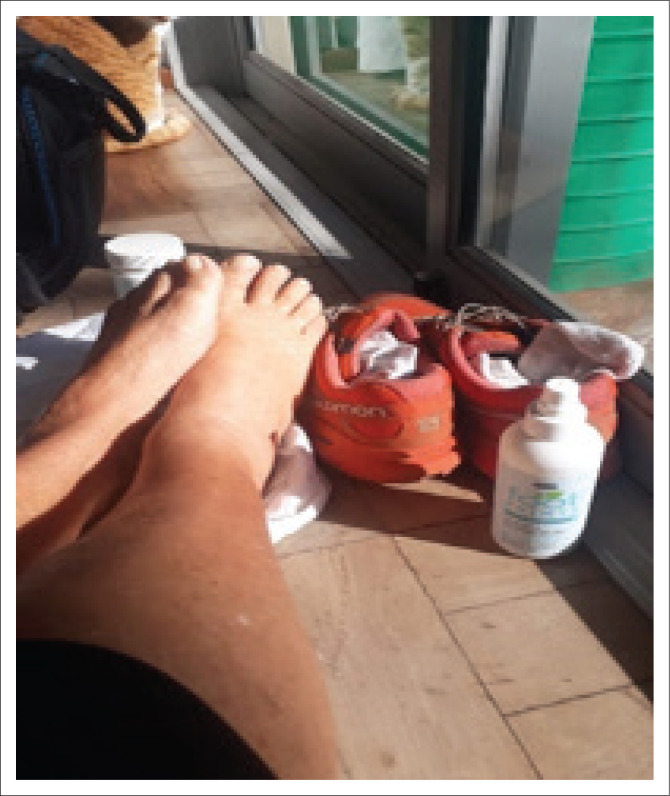
The participant photographed her feet after a run once she was resting and taking care of them.

The family physician who sent the worn-out running shoes (Figure 2) expresses her change to self as follows (Figure 7): ‘I thank my feet for resting on the earth. Thank the earth for gently supporting my rested and pampered feet. They deserve my undivided attention’.

In the last theme, participants appeared to feel present in their bodies and able to enjoy the moment, fully aware and without judgement, which personifies the feeling of mindfulness.

## Discussion

This study aimed to explore HCPs’ mindfulness experiences whilst working during the COVID-19 pandemic and gain insight into the impact of mindfulness on their sense of self and how they perceived their stressors. Furthermore, PhotoVoice aided the process of cognitive reappraisal by bringing awareness to their thoughts and emotions where words would not have done justice, assisting in creating a universality of experience and acceptance of the struggle of the time. This did not just change how HCPs related to their stressors but also how they saw themselves. Budig et al. (2018) confirmed this finding in their study when they too found that PhotoVoice could not just aid critical thinking and social awareness but transformed self-perception too. This study found that the HCPs could move from survival mode to consciously accept themselves without judgement for doing the best they could under the circumstances and finally caring for themselves, embracing their humanness. Johnson and Walsh (2021) explain this finding as mindfulness imbuing participants with a gentle curiosity about one’s thoughts and feelings, allowing for compassion and understanding towards self.

PhotoVoice was thus perceived as aiding the attitude of mindfulness by initiating the process of reappraisal with HCPs to articulate their experiences in an overwhelming situation. Reappraisal was observed in the mindfulness intervention, which afforded the HCPs a metacognitive awareness that required them to slow down, notice more and distance themselves from their perceptions to the extent that they could reinterpret their experiences. Their pictures clearly describe the process. An excellent example of this is the stark contrast between the initial pictures of drowning (Figure 4) to pictures of resting in nature (Figure 6) – moving from immersion to perspective; from a single, egocentric, isolated view to a broader, more encompassing universal view; from the mindless state of being busy and disconnected from themselves and overwhelmed by the environment to be able to take a breath, pause and permit themselves to connect more with themselves and nature. Johnson and Walsh (2021) hypothesise that this connection with the environment happens at two points when using PhotoVoice: (1) noticing and reflecting whilst taking the picture and (2) the collective production of knowledge when sharing pictures and engaging in discussions.

These findings revealed that by practising mindfulness, HCPs could move from a mindless state of automation to acceptance and faith. Faith in themselves made them feel more competent in handling their stressors, accepting that their distress was universal. Faith in a supernatural power helped them make sense of their suffering. The ‘non-acceptance’ factor, as identified by Chiodelli et al. (2018), is an inclination to judge negative emotions when individuals are not functioning at their peak. Non-acceptance can also be seen in the form of distraction to not deal with the issue at hand, such as using substances or devices. As the participants had described themselves initially, keeping oneself busy with work on autopilot could be part of the non-acceptance factor. Previous studies found that acceptance, especially self-acceptance, and validation of one’s feelings achieved through mindfulness led to better boundaries, enhanced coping and feelings of peace (Van Wietmarschen et al. 2018). Being present and accepting the situation for what it is appeared to be a juncture of change for our HCPs, who had to acknowledge this difficult time and accept that they would not always be coping with the demands of the situation. The realisation that most of them felt powerless and that this was a normal reaction to an abnormal situation appeared to be a form of relief. Ironically, by owning their limitations, they started to focus on what they could control and leave the rest to a supernatural power, which resulted in a sense of calmness. Chirico and Nucera (2020) acknowledge that spirituality, faith and prayer effectively reduce stress and increase health benefits, especially during the COVID-19 pandemic.

From an anxious and overwhelmed emotional state, participants who continued with the mindfulness practice started to speak of being kinder to themselves and taking better care of themselves. Self-compassion is an attitude of consideration towards oneself (Grise-Owens, Miller & Eaves 2016). To be self-compassionate, the HCPs had to learn to relate to themselves differently in choosing self-kindness and understanding instead of self-judgement and criticism (Neff 2003). Learning to be sensitive and respectful towards themselves facilitated being that way toward others in their care (Raab 2014). They had to acknowledge a sense of shared humanity in that many were suffering in this together instead of feeling isolated and trying to act like everything was normal, which led to feelings of incongruence (Neff 2003). Self-compassion is a critical skill for HCPs. It allows them to maintain their emotional sensitivity to patients and efficacy by alleviating their empathetic distress (Wasson, Barratt & O’Brien 2020).

Being empathetic towards others is vital for the work HCPs do but has been linked to the concept of compassion fatigue (ed. Figley 1995). Compassion fatigue is HCPs’ reduced ability to care and be present because of the overwhelming emotions resulting from being exposed to the pain of others over long periods. It is one of the components of burnout (Raab 2014). Paradoxically, self-compassion is linked with qualities of resiliency against stress, burnout and emotional exhaustion (Raab 2014). Hence, the ability to take one’s own experiences and emotions into account and connect to them, without any judgement and with a sense of curiosity, helps HCPs cope with the uncertainty of the time.

When the participants were able to disengage from their states of autopilot and be more present, they slowed down and were more open to noticing nature and the context around them. Like other studies, this study found that mindfulness enhanced the awareness of self–nature interconnectedness by intensifying the experiences within the natural environment (Liu & Valente 2018). This could be one of the reasons why the researchers received so many pictures of nature under the theme of self-compassion. The use of nature as a calming, therapeutic factor and the harmony felt with it directly contrasted with the metaphor of natural disasters – ‘like I’m caught in a whirlwind’ – as described by how the participants felt under the theme of overwhelm. The use of open spaces also contrasted with the feeling of initially feeling trapped and out of control, as because of COVID-19 and the subsequent lockdowns and quarantines, participants could have felt closed in and closed off. These images could be symbols of hope and courage that the HCPs required not just to survive but to thrive in this time. Badanta et al. (2021) had similar findings in a paper titled ‘a picture says a thousand words’. They commented on the importance of seeing the world through the eyes of the HCPs, particularly during these moments of strife, and how PhotoVoice highlighted feelings of empowerment and a sense of achievement through creative expression.

Based on the findings of this study, a brief online MBI was impactful enough to depict reappraisal of the stressors of COVID-19 such that HCPs felt calmer, more competent and more compassionate. Subsequently, using an MBI as a stress-reduction technique is highly recommended. Furthermore, PhotoVoice methodology is recommended with MBIs for future studies and mindfulness courses. PhotoVoice did not just aid the understanding of the process of imbuing mindfulness but assisted in the attitude of mindfulness and curiosity and added a sense of objectivity that aided positive reappraisal coping.

## Strengths and limitations

A significant strength of this study was that, even though some HCPs had high stress levels and participants were of different backgrounds, positive changes were noted in the pictures from the participants’ self-reports. This points to the notion that, despite their differences, a group of practising frontline HCPs were able to rise above a difficult situation that was overwhelming and could benefit from an MBI no matter their background, ethnicity or social standing, thus making an MBI an excellent option for stress reduction for HCPs both in training and practice.

The researchers found that the use of PhotoVoice in this study facilitated a deeper understanding of HCPs’ perspectives and experiences. This novel study illustrates how photographs can provide a visual representation into the process of imbuing mindfulness and insight into what HCPs gained from practising mindfulness during the COVID-19 pandemic. In terms of the experience of the PhotoVoice process itself, many of the participants reflected on the course of choosing a picture and reported finding it difficult initially. They observed that they first had to quieten down to become aware of their feelings and then find a picture that encapsulated these feelings. However, when they did find that picture, there was a sense of catharsis. This became part of the emotional regulation process that was already being encouraged with the mindfulness practice. Many participants observed that PhotoVoice was an exercise in mindfulness. Mahalingam and Rabelo (2019) similarly observed the use of PhotoVoice to describe mindfulness exercises and the subsequent change in behaviour as mind-altering pedagogies that enhanced transformation.

In terms of the study limitations, it is acknowledged that, despite nurses being amongst the critical frontline workers during the pandemic, they did not participate in the study mainly because of the reasons provided in our research methods section. Therefore, the study recommends that future research on this subject target nurses as participants.

## Conclusion

It is critical to improve HCPs’ overall well-being, especially in chronic stress situations such as a global pandemic. Awareness and present-moment focus with the attitude of curiosity instead of judgement, which is the very definition of mindfulness, led to acceptance, a sense of perspective and self-compassion. Being mindful, being present to their experiences and considering their own needs and emotions helped HCPs be calmer, more self-assured and more connected. It was not possible to change the demands of this time. However, through mindfulness, it appeared that the HCPs could reframe their perspectives and cope with the set of challenges they faced with more resilience and healthier coping mechanisms. Their perspectives changed from mindless reaction to a mindful ability to respond. Their perception and responses changed by bringing awareness to how the HCPs saw this challenging time, which was enhanced by using PhotoVoice methodology. This had a profound impact on their sense of well-being and their ability to cope with the disruptions of a global pandemic.
